# Nurse Managers’ Emotional Intelligence and Effective Leadership: A Review of the Current Evidence

**DOI:** 10.2174/1874434601812010086

**Published:** 2018-05-31

**Authors:** Panagiotis E Prezerakos

**Affiliations:** Laboratory of Integrated Health Care, Nursing Department, University of Peloponnese E & St. Valioti and Plateon Str., 23100, Sparta, Greece

**Keywords:** Emotional Intelligence, Leadership, Nurses, Nurse managers, Nurse leaders, Calcium

## Abstract

**Background:**

Emotional Intelligence has made a significant contribution to effective leadership, becoming one of the key characteristics of leaders.

Objective: The aim of the present study was to review qualitative and quantitative studies concerning Emotional Intelligence of nurse leaders and the evidence-based composition of their results.

**Method:**

A search was performed in the electronic databases (Pubmed, Scopus and CINAHL) for articles, which were published in the period 2000-2017 in English or Greek. Eleven studies met the inclusion criteria, of which 10 were quantitative and one was qualitative.

**Results:**

The results suggested that Emotional Intelligence is a useful tool for nurse leaders and contributes decisively to the achievement of effective management in healthcare.

**Conclusion:**

It is necessary for nurses to improve their social and emotional skills because of the particular nature of the nursing profession, which places the healthy or weak person at its center.

## 1 BACKGROUND

“Emotional Intelligence: Why It Can Matter More Than IQ” was the title of Goleman's book [1] which made the concept of Emotional Intelligence (EI) widely known and contributed significantly to the expansion of research in this field. Salovey& Mayer [2] define EI as, “the subset of social intelligence that involves the ability to monitor one's own and others' feelings and emotions, to discriminate among them and to use this information to guide one's thinking and actions”. Emotionally intelligent individuals are able to use, understand and manage their feelings in a way that benefits themselves and others [3]. Studies conducted with children, adolescents and adults highlight the beneficial effect of EI on the happiness of the individual [4], emotional control [5], decision making [6], job satisfaction [7], occupational commitment [8] and Human Resources Management [9].

Recent theories emphasize that effective leadership is affected by the personality of the leader, the general conditions in the workplace and the quality characteristics of employees [10].More specifically, Skinner and Spurgeon [11] note that health leadership involves understanding and communicating with a wide variety of individuals in a number of different situations and not just focusing on work results and rational processes. From this perspective, the fact that the EI has made a significant contribution to effective leadership becomes one of the key characteristics of leaders. As Cummings and his colleagues [12] support, emotionally intelligent nurses with an administrative position inspire emotions, passion and motivation helping thus to achieve goals that might otherwise have not been conquered.

The aim of the present study was to systematically review qualitative and quantitative studies concerning EI of nurse leaders and the evidence-based composition of their results. In particular, it was attempted to highlight the reasons why EI is important for achieving effective nursing leadership

## 2 MATERIALS AND METHODS

**Fig. (1):**
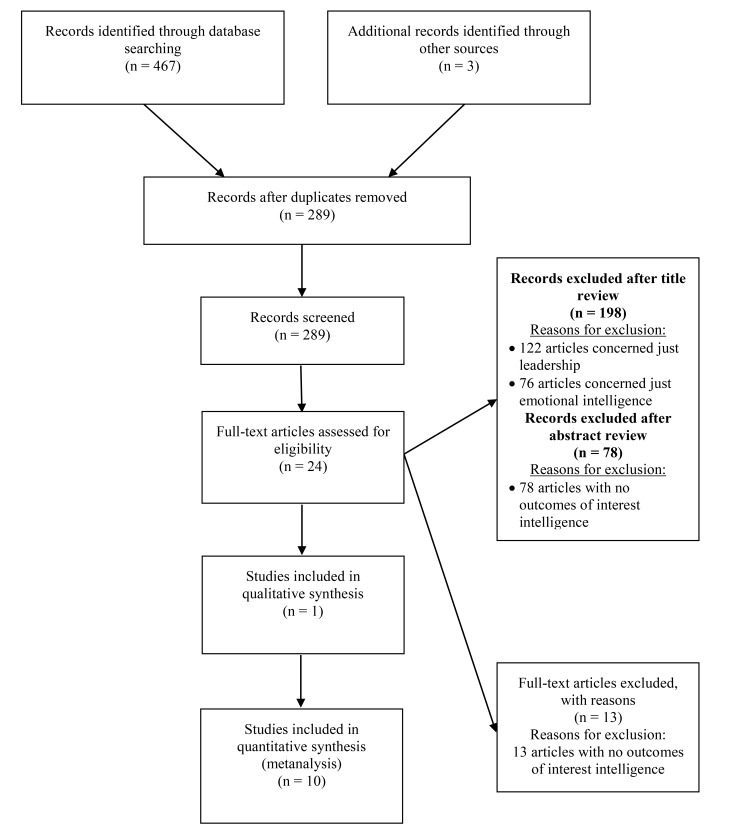
The entire sample was then critically analyzed in order to outline the reasons why EI is important for achieving effective nursing leadership in the health sector.

A search was performed in the electronic databases (Pubmed, Scopus and CINAHL) for articles, which were published in the period 2000-2017 in English or Greek. The literature review was carried out from September to October 2017. The keywords used were: Emotional Intelligence, Leadership, and Nursing. The inclusion criteria that were applied included (a) the publication date had to be between 2000 and 2017, (b) the languages were Greek or English, (c) both qualitative and quantitative studies were included, and (d) the sample of studies reviewed included only nurses. In order to achieve a final list of related studies, a broad literature search was conducted to identify abstracts that met the inclusion criteria. The titles and abstracts were printed, duplicates were discarded and the remaining abstracts were examined using the inclusion/exclusion criteria (Fig. **1**).

## 3 RESULTS

The literature review included 470 articles. Of these, 300 abstracts were evaluated by the author with regard to their compatibility with the inclusion criteria in the analysis (Flowchart). Finally, eleven (11) studies met the inclusion criteria Table **1**, of which seven (7) were conducted in the United States of America [13-19], one (1) in Turkey [20], one (1) in Australia [22] and one (1) in Canada [23]. Only one (1) of the selected studies was qualitative [22], in which the researchers used focus groups to investigate the research question. In the remaining studies [13-21, 23] data collection was carried out using structured questionnaires. With regard to the sampling method, only two studies [20, 23] were randomly sampled.

Three of the eleven studies included in the review explored the leadership style applied by emotionally intelligent nurses holding an administrative position [14, 17, 18]. The critical analysis of their results revealed the positive correlation between the Emotional Inelegance and the transformational leadership in which the leader works with subordinates to increase their motivation and promote their commitment to the organization [24]. In two studies [17, 18], there was a statistically significant positive correlation of the EI with the outcome of the leadership on the extra effort, effectiveness and satisfaction of EI, Spano-Szekely and her colleagues [17] underline the negative relationship between the EI and the laissez-faire leadership, in the course of which, according to Harms and Credé [25], leaders are avoiding decision making, are reluctant to take action and their role is typical. In contrast, in the study conducted by Tyczkowski and her associates [18], the above negative relationship is not confirmed. Furthermore, no significant correlation observed between EI and transactional leadership styles [18].

Erkutlu and Chafra’s [20] study, on a sample of 910 nurses, revealed that high nurse leaders’ EI have a strong positive impact on the pro-activity and empowerment of the team [23]. The results of Munro’s study [16] pointed out that the high EI of nurses performing administrative tasks is associated with increased patient satisfaction with the care provided. In addition, EI along with resilience, self-awareness and understanding of other clinical disciplines were revealed as important qualifications for nurse managers. Moreover, emotionally intelligent leadership, through the skills of good emotional management, promotes well-being at the workplace [21]. Regarding the relationship of EI and demographic characteristics, it was noticed that nurses, who exercised administrative duties and had not received any specialization, showed modest emotional intelligence in contrast to their colleagues, who were specialized [13]. In addition, nurses, who held a post-graduate degree in nursing science, had enhanced their skills in the proper use of emotions, compared to nurses who had specialized in another field [15]. Equally important was the experience in management positions, as managers with less than two years of experience experienced the less successful use of their feelings than those with longer seniority in management positions [15].

## 4 DISCUSSION

The results of this study suggest that EI is a useful tool for nurse leaders and contributes decisively to the achievement of effective management in healthcare. The nature of the nursing profession itself, aimed at health promotion, disease prevention and care of physically and mentally ill and disabled people of any age [26], requires nurses to be emotionally intelligent in order to respond to their multifarious duties. The anthropocentric nature of nursing requires Emotional Intelligence as a high-level skill that contributes to effective patient centered care [27]. More specifically, with regard to the exercise of management functions, EI becomes an important “virtue” in the hands of nurse-leaders who must be equipped with skills to successfully meet the growing demands of the modern health care system. Management skills, such as negotiating resources, building trust relationships, encouraging partnership development, and making evidence-based decisions, require a strong foundation of perceiving, using, understanding and managing feelings [13]. Analysis of a number of the studies selected [14, 17, 18] revealed that EI was associated with the exercise of the transformational leadership. According to Bass and Avolio [28] transformational leadership refers to the idealized influence that the leader exercises on his/her subordinates, the feeling of pride which he/she inculcates as well as the feeling of safety and confidence that they can cope with the organization’s goals and vision. The high ability to understand, regulate and manage emotions that transformational leaders have, contributes decisively to the cultivation of corresponding skills in their followers [29]. EI’s strong association with transformational leadership is not present only in the health sector but also in a variety of other organizations [30-33]. Barling, Slater and Kelloway’s study [34], which deals with a large pulp and paper industry, revealed a positive correlation between the EI and the three aspects of transformational leadership: idealized influence, inspirational motivation, and individualized consideration. Similarly, in Sivanathan and Fekken’s [35] study, in the university community, established that high levels of emotional intelligence were associated with transformational leadership model. Furthermore, the same analysis revealed that the emotionally intelligent management “triggers» proactivity [20], the team empowerment [20, 23], patient satisfaction of care provided [16], wellbeing at work [21] and contributes decisively to pay extra effort on the part of subordinates, efficiency and job satisfaction [17, 18]. Developed emotional skills of leaders and followers, are instrumental in achieving a healthy work environment, not only in the healthcare field but also in ministries, public services, security bodies, private companies, industries, schools, reinforce organizational commitment [36-39], job satisfaction [36-38, 40, 41] and wellbeing in the workplace [38].

## CONCLUSION

EI is important for achieving effective leadership in healthcare organizations and contributes decisively to their good-functioning and successful operation. Individuals have the ability to identify and experience a wide range of emotions in everyday life. However, some of them are not able to use, understand and manage these emotions. That fact suggests that it is necessary to improve their social and emotional skills. As regards nurses, at every level, the above need is considered imperative because of the particular nature of the nursing profession, which places the healthy or weak person at its center. By implementing social and emotional learning programs, nurses can acquire knowledge, attitudes and skills that are necessary for understanding and managing emotions, achieving positive goals, and maintaining positive relations and accountable decisions.

## Figures and Tables

**Table 1:** An overview of studies’ characteristics, aims and main findings

**Authors (Year of Publication)**	**Title** **Type of Research** **-Number of participants)**	**Country**	**Aim**	**Results**
Spano-Szekely, Griffin, Clavelle, Fitzpatrick, (2016) [17]	Emotional Intelligence and Transformational Leadership (TL) in Nurse Managers (Cross-sectional, quantitative, 148 nurse managers)	USA	To determine the relationship between EI and TL in frontline acute care NMs.	EI was significantly positively correlated with TL and outcome measures of extra-effort, effectiveness, and satisfaction and significantly negatively correlated with laissez-faire leadership
Tyczkowski *et al.*, (2015) [18]	Emotional Intelligence (EI) and Nursing Leadership Styles Among Nurse Managers (Cross-sectional, quantitative, 142 nurse managers)	USA	To examine the relationships among education, leadership experience, emotional intelligence and transformational leadership of nurse managers.	Statistically significant positive relationships were noted between EI and transformational leadership and the outcomes of leadership (extra effort, effectiveness, and satisfaction). No statistically significant relationships were noted between EI and transactional or laissez-faire leadership styles.
Leggat & Balding, (2013) [22]	Achieving organizational competence for clinical leadership: The role of high performance work systems (Qualitative, 28 clinicians and clinician managers)	Australia	To present the results of a qualitative study with clinicians and clinician managers to gather opinions on the appropriate content of an educational initiative being planned to improve clinical leadership in quality and safety among medical, nursing and allied health professionals working in primary, community and secondary care.	Only four individual factors, comprising emotional intelligence, resilience, self-awareness and understanding of other clinical disciplines, were identified as being important for clinical leaders.
Lucas, Spence- Laschinger & Wong, (2008) [23]	The impact of emotional intelligent leadership on staff nurse empowerment: the moderating effect of span of control (Cross-sectional, quantitative, 230 nurses)	Canada	To test a model linking nurses perceptions of their nurse manager s emotionally intelligent leadership style and nurses structural empowerment, and the impact of nurse manager span of control (number of direct reports) on the emotional intelligence/empowerment relations	Span of control was a significant moderator of the relationship between nurses’ perceptions of their managers’ emotionally intelligent behavior and feelings of workplace empowerment.
Moss, Ritossa, & Ngu, (2006) [19]	The effect of follower regulatory focus and extraversion on leadership behavior: The role of emotional intelligence. (Cross-sectional, quantitative, 66 pairs of nurses and their supervisors)	USA	Emotional intelligence should enhance the capacity of managers to adapt their leadership style.	Emotional intelligence might enhance the capacity of managers to adapt their leadership style appropriately, but only in some contexts
Spagnuolo *et al.*, (2014) [21]	Emotional Leadership: a survey on the emotional skills expressed by nursing management (Cross-sectional, quantitative, 130 managers, head nurses and nurses)	Italy	This study investigates knowledge about the emotional leadership and emotional competence in nursing management.	It is essential for managerial roles, be aware and able to manage their own and others' emotions to generate wellbeing at work.
Ohlson & Anderson, (2014) [13]	Ability emotional intelligence of nurse managers in the Midwestern United States (Cross-sectional, quantitative, 87 nurse managers)	USA	To describe the emotional intelligence (EI) and examine the corresponding demographic characteristics of front-line Nurse Managers in acute care settings	What is interesting is the significant difference in EI between nurses who are certified in any specialty versus those who are not certified at all.
Echevarria, Patterson & Krouse, (2016) [14]	Predictors of transformational leadership of nurse managers (Cross-sectional, quantitative, 148 nurse managers)	USA	To examine the relationships among education, leadership experience, emotional intelligence and transformational leadership of nurse managers.	Statistically significant relationship was found between emotional intelligence and transformational leadership (r = 0.59, P < 0.001) explaining 34% variance in transformational leadership.
Erkutlu &Chafra, (2012) [20]	The impact of team empowerment on proactivity The moderating roles of leader’s emotional intelligence and proactive personality (Cross-sectional, quantitative, 910 certified nurses in 82 teams from 12 university hospitals)	Turkey	To investigate the relationship between team empowerment and team proactivity and the moderating roles of a team leader’s emotional intelligence (EI) and a team member’s proactive personality	Proactivity is positively associated with team empowerment. In addition, team leader’s EI and team members’ proactive personality influence the relationship between team empowerment and team proactivity. Specifically, teams exhibit the highest proactivity when team leaders’ EI and team members’ proactive personality are high.
Prufeta, (2017) [15]	Emotional Intelligence of Nurse Managers (Cross-sectional, quantitative, 38 nurse managers)	USA	To determine the level of emotional intelligence (EI) among nurse managers (NMs) in an academic medical center and determine the relationship of EI and demographic variables.	Mean EI scores among NMs were average. Nurse managers with less than 2 years of experience had statistically significant lower busing emotions branch score and strategic EI. Nurse managers with a master’s_ degree in nursing scored significantly higher in using emotions branch score than did those with a master’s degree in a related field.
Munro, (2011) [16]	Nurse Manager Emotional Intelligence as a Predictor to Registered Nurse Job Satisfaction and RN Perceptions of the Practice Environment and the Relationship to Patient, Nursing and Hospital Outcomes (Cross-sectional, quantitative, 38 nurse managers & 659 RNs)	USA	To investigate if the level of Nurse Manager (NM) emotional intelligence (EI) predicted registered nurse (RN) job satisfaction and RN perceptions of the practice environment.	NM EI was significantly correlate and patient satisfaction with nursing care.

## LIST OF ABBREVIATION

EI: = Emotional Intelligence
